# A Case of Acute Promyelocytic Leukemia With Retinal Hemorrhages Beneath Internal Limiting Membrane During Clinical Remission

**DOI:** 10.7759/cureus.13387

**Published:** 2021-02-17

**Authors:** Marwan M Balubaid, Abdullah S Alqahtani

**Affiliations:** 1 Internal Medicine, King Abdulaziz Medical City, Jeddah, SAU; 2 Ophthalmology, King Abdulaziz Medical City, King Saud bin Abdulaziz University for Health Sciences, Jeddah, SAU

**Keywords:** retinal hemorrhage, leukemia, aml, internal limiting membrane hemorrhage, leukemic retinopathy

## Abstract

Leukemia is a systematic cancer of the blood and blood-forming tissues that affects multiple organs. Acute myeloid leukemia (AML) is the most common type of leukemia that affects adults. Ophthalmic manifestations of leukemia could be observed in both acute and chronic leukemias. Around 35.4% of leukemia patients present with leukemic retinopathy. In this report, we discuss the case of a patient who was diagnosed with acute promyelocytic leukemia (APL) and went on to develop leukemic retinopathy during chemotherapy.

A 35-year-old male was diagnosed with APL and received induction therapy with daunorubicin, and all-trans retinoic acid (ATRA) in a seven + three regimen. During the remission phase, he presented with a complaint of decreased vision of the right eye for about three weeks after the initiation of the therapy. On examination, the best-corrected visual acuity (BCVA) was found to be 6/60 in the right eye and 6/6 in the left eye. Fundus examination showed intraretinal hemorrhages in the posterior pole of both eyes. Fundus photography of the right eye showed resolved macular bleeding, temporal retinal bleeding, fresh inferonasal, and supraoptic retinal hemorrhage. For the left eye, however, it showed a small hemorrhagic spot temporal to the macula. Optical coherence tomography (OCT) was performed on both eyes, which showed sub-inner limiting membrane (sub-ILM) hemorrhage in the right macula with normal OCT of the left eye.

There are multiple reported ocular manifestations of leukemic retinopathy including flame-shaped hemorrhage, cotton wool spots, and Roth spots. In patients with APL, thrombocytopenia and intracranial hemorrhage are the proposed underlying mechanism of retinal hemorrhage. Terson’s syndrome, which is an intracranial hemorrhage with associated retinal hemorrhage, has been reported to occur during ATRA induction therapy. Sub-ILM hemorrhage is relatively uncommon, and it has been associated with multiple primary pathologies such as Valsalva retinopathy, Terson’s syndrome, and bleeding dyscrasias.

Retinal hemorrhage is a serious complication in leukemic patients; it could be the presenting complaint or even manifest after the initiation of therapy. Early detection and frequent follow-ups are crucial for its management.

## Introduction

Leukemia is a systematic cancer of the blood and blood-forming tissues, and it affects multiple organs. Based on the form of the abnormal leukocytes, duration of symptoms, and how quickly they leave the bone marrow and enter the blood, leukemia is further classified into acute myeloid leukemia (AML), acute lymphoblastic leukemia (ALL), chronic myeloid leukemia (CML), and chronic lymphocytic leukemia (CLL). AML is a complex disorder that arises in a malignantly hematopoietic stem cell and subsequently acquires successive genomic alterations, eventually developing into clinically overt disease. AML is the most common type of leukemia that affects adults [1].

The eye is considered to be a distinct site where the impact of leukemia on nerves and blood vessels could be directly observed. According to previous studies, both acute and chronic leukemia could have ocular involvement. However, acute leukemia is significantly more common than chronic leukemia [2]. Ocular manifestations of leukemia could be due to either direct infiltration of neoplastic cells or from chemotherapy, leading to hematologic abnormalities like anemia, hyperviscosity, and thrombocytopenia [3]. Uncommonly, the retina can be affected in leukemic patients due to a similar mechanism [4].

Leukemic retinopathy is a term used to describe the retinal manifestations of anemia, thrombocytopenia, and hyperviscosity leading to hemorrhage, rather than leukemic infiltration [4]. Around 35.4% of leukemia patients present with leukemic retinopathy [5]. In this study, we present a case of acute promyelocytic leukemia (APL) that developed sub-internal limiting membrane (sub-ILM) hemorrhage after undergoing all-trans retinoic acid (ATRA) therapy.

## Case presentation

**Figure 1:**
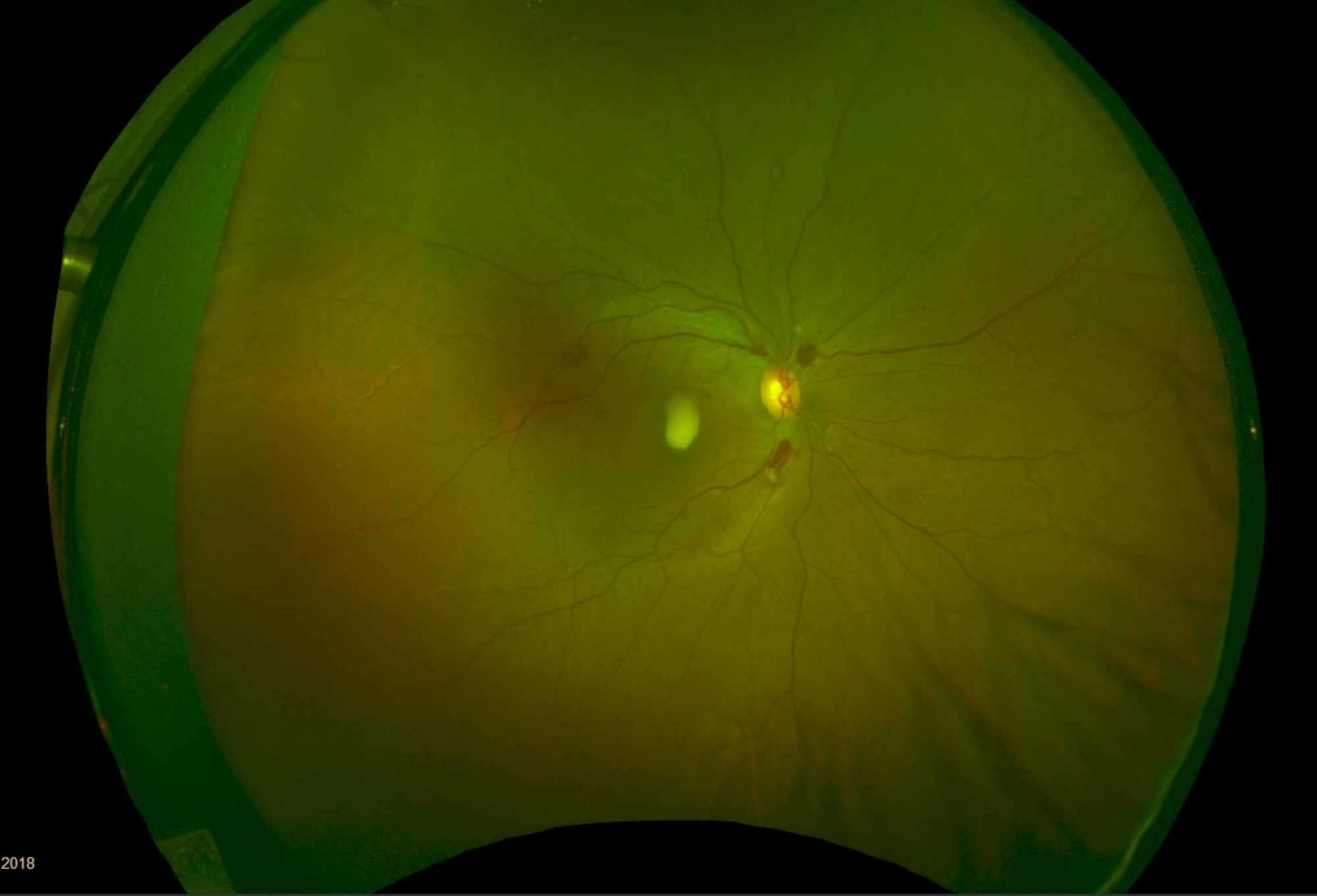
Fundus photo of the right eye The image shows resolved macular bleeding, temporal retinal bleeding, and fresh inferonasal and supraoptic retinal hemorrhage

**Figure 2:**
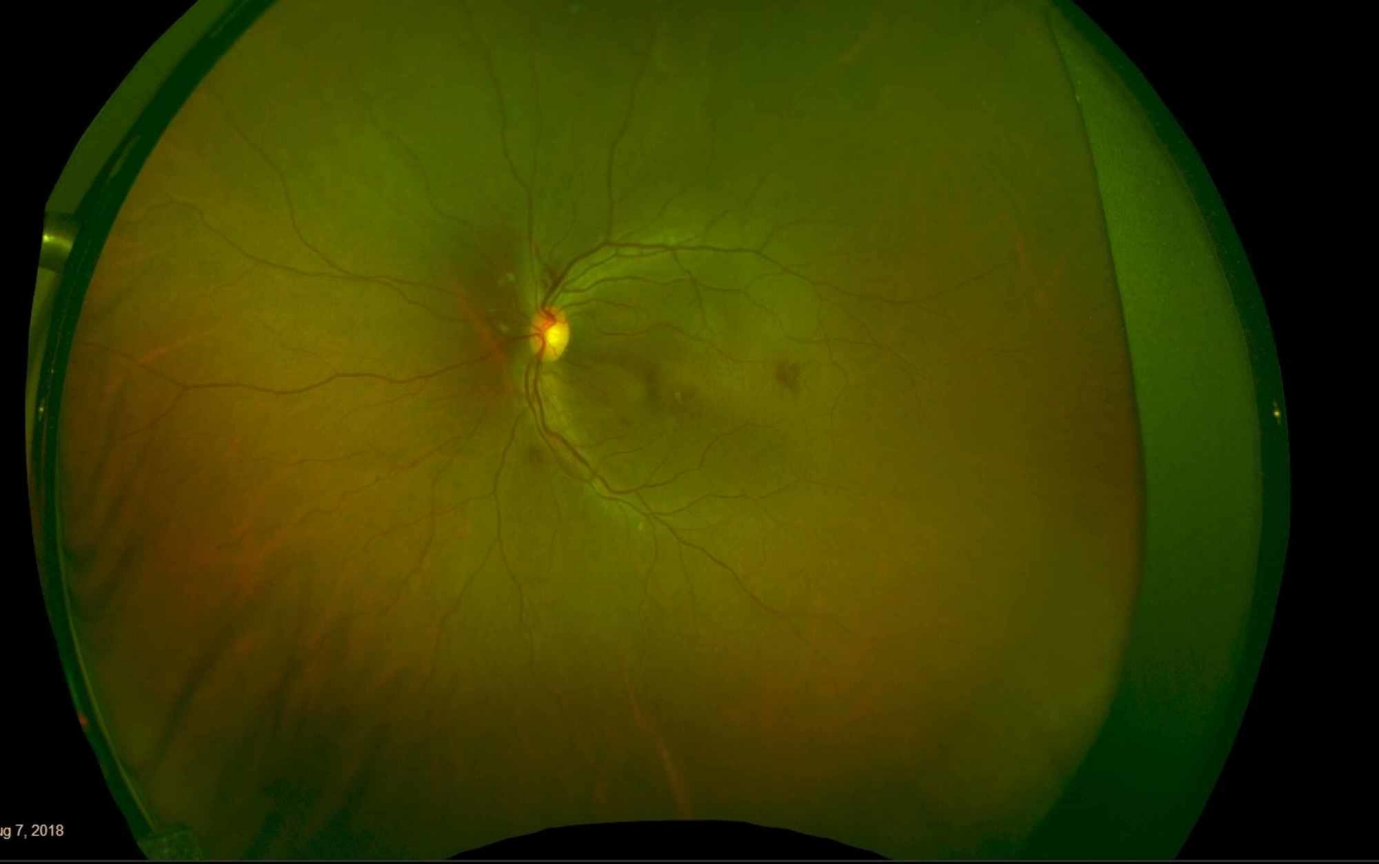
Fundus photo of the left eye The image shows a small hemorrhagic spot temporal to the macula

**Figure 3:**
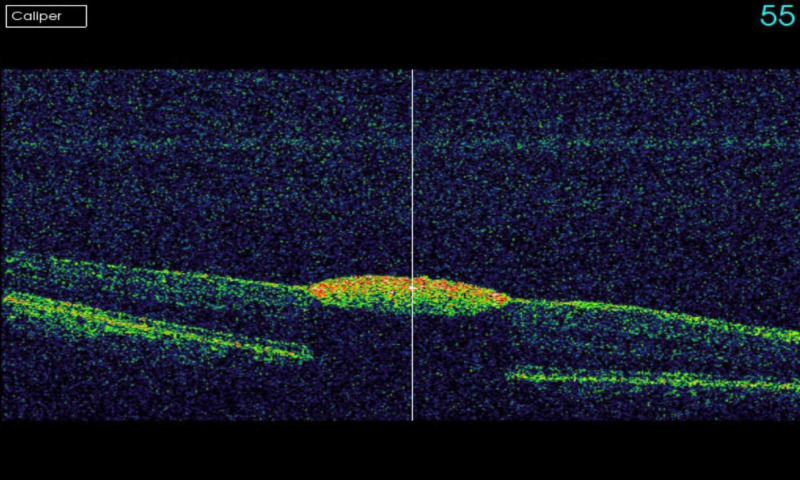
OCT of the right eye The image shows sub-inner limiting membrane (sub-ILM) hemorrhage in the right macula OCT: optical coherence tomography

**Figure 4:**
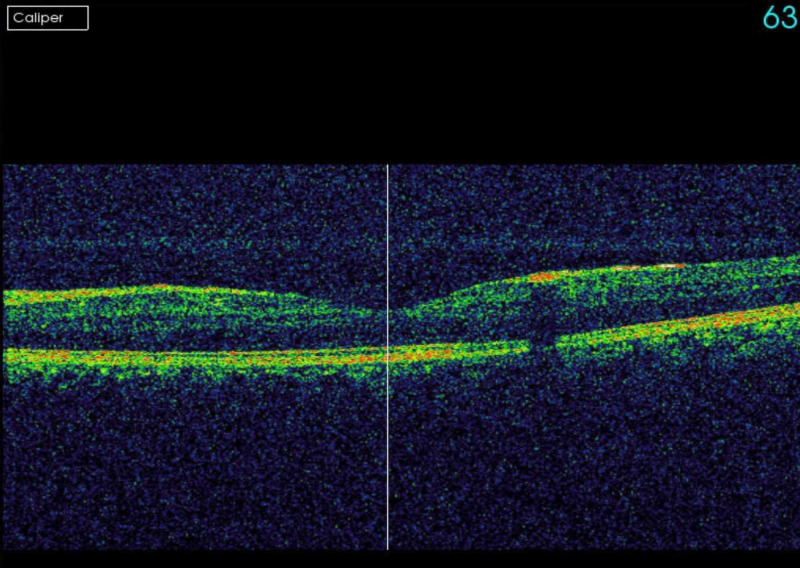
OCT of the left eye The image shows no sign of abnormality OCT: optical coherence tomography

A 35-year-old male with no previous medical issues presented to another hospital with the main complaint of fever (38 °C), fatigue, melena, hematuria, and thrombocytopenia. Complete blood count (CBC) showed leukocytes of 20,000 mm^3^, platelets of 46,000 mm^3^, and an international normalized ratio (INR) of 1.39. The patient underwent a bone marrow biopsy, which showed granulopoiesis (active, dominated by promyelocytes in 50% of the nucleated marrow cells), and he was diagnosed with APL. Fluorescence in situ hybridization (FISH) for PML-RARA was ordered and came back negative. He received induction therapy with daunorubicin, and ATRA in a seven + three regimen in August 2018. The patient was referred to our hospital for the continuation of care. After a month of undergoing the treatment regimen, he presented to the ER with a complaint of neutropenic fever (38.2 °C) and decreased vision in the right eye. On examination, the best-corrected visual acuity (BCVA) was found to be 6/60 in the right eye and 6/6 in the left eye. The pupils were normal and briskly reacting to the light with no relative afferent pupillary defect (RAPD). An anterior segment exam was within the normal limit: it showed clear cornea, and the anterior chamber (AC) was deep and quiet, with normal iris in both eyes. Fundus examination showed intraretinal hemorrhages in the posterior pole of both eyes. There was a yellowish lesion in the center of the macula around X1.3-disc diameter of the right eye. Fundus photo of the right eye showed resolved macular bleeding, temporal retinal bleeding, and fresh inferonasal and supraoptic retinal hemorrhages. For the left eye, however, it showed a small hemorrhagic spot temporal to the macula (Figure 1, Figure 2). Optical coherence tomography (OCT) was performed for both eyes, and it showed sub-ILM hemorrhage in the right macula with normal OCT of the left eye (Figure 3, Figure 4).

The patient was only managed expectantly and instructed to return for a follow-up visit after two weeks. At the follow-up visit, his vision was found to be improved: the BCVA of the right eye was 6/7.5, and that of the left eye was 6/6.

## Discussion

The ocular manifestations of leukemic retinopathy are diverse. Previously reported findings have included flame-shaped hemorrhage, cotton wool spots, Roth spots (white-centered hemorrhage), retinal venous tortuosity, and neovascularization [2]. Leukemic retinopathy is commonly seen at the posterior pole or may extend into vitreous or subretinal spaces [4].

APL is a distinct subtype of AML that is characterized by a specific translocation t(15;17) forming the PML-RARA gene that drives abnormal promyelocyte proliferation [6]. Clinically, significant coagulopathy is found to be present in 70-80% of APL patients upon disease diagnosis [7]. Thrombocytopenia and intracranial hemorrhage are both suggested to be the underlying mechanism of APL progression to retinal hemorrhage. Intracranial hemorrhage, especially subarachnoid hemorrhage (SAH) that is associated with retinal hemorrhage, is called Terson’s syndrome [8].

ATRA is a vitamin A derivative that has recently been reported to promote differentiation of leukemic promyeloblasts into mature granulocytes, resulting in the bone marrow and cytogenetic complete remission rate of 64-96% [9]. Abu el-Asrar et al. have reported a case of bilateral intraocular hemorrhage in association with cerebral bleeding in a patient with APL during ATRA induction therapy [10].

Sub-ILM hemorrhage is a relatively uncommon phenomenon; it is usually associated with a variety of etiologies and often leads to the loss of visual acuity [11]. Valsalva retinopathy, a sudden increase in pressure of the intraocular veins, Terson’s syndrome, and bleeding disorders are different mechanisms proposed to cause sub-ILM hemorrhage [12]. The proposed mechanism of the latter suggests that a combination of severe anemia and thrombocytopenia may result in reduced endothelial cell integrity of the retinal vasculature and reduced coagulability, allowing the blood to leak through the endothelial barrier [11].

In our case, the patient developed sudden visual loss due to sub-ILM hemorrhage after three weeks of starting ATRA therapy (remission phase), which is a rare occurrence as per the literature. Besides, there was no sign of any intracerebral hemorrhage or intracranial hypertension, which has been reported to have an association with retinal hemorrhage. This case highlights the importance of retinal examination that clinicians should perform in leukemic patients even after the initiation of ATRA therapy.

The treatment for leukemic retinopathy does not involve any direct therapy. Chemotherapy, immunotherapy, and radiotherapy, however, are usually implemented to manage the underlying systemic cause [1]. In 2018, Lyu et al. suggested that expectant management should be the first-line treatment of choice due to the higher risk of bleeding and infection in leukemic patients [13].

## Conclusions

Retinal hemorrhage is a serious complication in leukemic patients; it could be the presenting complaint or it may even manifest after the initiation of therapy. It could result from direct leukemic pathological infiltrates or secondary anemia, thrombocytopenia, or hyperviscosity. Early detection and frequent follow-ups are crucial for its management. This case highlights the importance of routine eye exams that ophthalmologists should perform in leukemic patients.
